# Non-contrast myocardium blood flow: consideration of technical differences between 4D Time-SLIP using tagging aortic root and FAIR

**DOI:** 10.1186/1532-429X-18-S1-P18

**Published:** 2016-01-27

**Authors:** Mitsue Miyazaki, Xiangzhi Zhou, Tsutomu Hoshino, Kenichi Yokoyama, Rieko Ishimura, Toshiaki Nitatori

**Affiliations:** 1 MRI, Toshiba Medical Research Institution, USA, Venon Hills, IL USA; 2 Radiology, Kyorin University, Tokyo, Japan

## Background

A non-contrast 4D Time-Spatial Labeling Inversion Pulse (Time-SLIP) technique [1, 2] (3D acquisition and time) has been developed to investigate myocardial bloodflow on healthy volunteers without administration of contrast materials. The technical differences are discussed between our 4D Time-SLIP using tagging aortic root and Flow-sensitive Alternating inversion recovery (FAIR) using a globally IR tagging pulse and control pulse in imaging plane [3, 4].

## Methods

The non-contrast 4D Time-SLIP technique was applied on eight healthy volunteers to image myocardium blood flow at 1.5T. The Time-SLIP sequence has a tagging block with a non-selective inversion recovery (non-sel-IR) pulse and a spatially selective inversion recovery (sel-IR) and a control block with applying only the non-sel-IR pulse. Both tagging and control were followed by a 3D bSSFP readout. The tagging plane was placed at the proximal ascending aorta and imaging slab was at mid-ventricle. In the tag acquisition, the non-sel-IR pulse inverts the magnetization (-Mz), and the spatially sel-IR pulse immediately inverts back the blood magnetization at the proximal ascending aorta (+Mz). In contrast, the control acquisition applies only the non-sel-IR pulse, which inverts the longitudinal magnetization to -Mz and experiences exponential T1 relaxation. The complex subtraction between tag and control depicts only the tagged blood flowing into the myocardium and the background signal was canceled out. Each 3D tag/control acquisition was performed within a breathhold and this was repeated for multiple TIs. The time resolved 3D short axis myocardial images were registered and segmented for the visualization of myocardial signal changes caused by the tagged blood along the TI. Perfusion curves were also generated to identify the perfusion peaks.

## Results

Our 4D Time-SLIP results showed that basal to apical blood flow can be observed in all volunteers using the 4D Time-SLIP technique. At the mid-ventricle, the blood flow reaches peak about 200-400 ms after tagging the aortic root blood, and then returned to baseline. This was observed across all volunteers. In comparison, 4D Time-SLIP is a 3D acquisition with time by tagging the blood at aortic root; whereas FAIR is a 2D single slice technique with the blood tagged in a global scale. Furthermore, the myocardial perfusion using FAIR was reported to have a signal tailing effect which did not return to baseline after the peak flow [4]. Our Time-SLIP method showed no tailing signal effect after the peak flow, which may provide useful information for various myocardial perfusion/flow defects.

## Conclusions

Our preliminary results indicate that the 4D Time-SLIP technique permits obtaining non-contrast myocardium perfusion images with high temporal resolution, and may potentially differentiate diseased myocardium from the normal one.
